# Latrophilin’s Social Protein Network

**DOI:** 10.3389/fnins.2019.00643

**Published:** 2019-06-26

**Authors:** J. Peter H. Burbach, Dimphna H. Meijer

**Affiliations:** ^1^Department of Translational Neuroscience, UMCU Brain Center, University Medical Center Utrecht, Utrecht, Netherlands; ^2^Department of Bionanoscience, Kavli Institute of Nanoscience Delft, Delft University of Technology, Delft, Netherlands

**Keywords:** latrophilin, synapse biology, developmental neuroscience, neurodevelopmental disorders, interaction networks

## Abstract

Latrophilins (LPHNs) are adhesion GPCRs that are originally discovered as spider’s toxin receptors, but are now known to be involved in brain development and linked to several neuronal and non-neuronal disorders. Latrophilins act in conjunction with other cell adhesion molecules and may play a leading role in its network organization. Here, we focus on the main protein partners of latrophilins, namely teneurins, FLRTs and contactins and summarize their respective temporal and spatial expression patterns, links to neurodevelopmental disorders as well as their structural characteristics. We discuss how more recent insights into the separate cell biological functions of these proteins shed light on the central role of latrophilins in this network. We postulate that latrophilins control the refinement of synaptic properties of specific subtypes of neurons, requiring discrete combinations of proteins.

## Introduction

Brain circuits function by virtue of precise connections between nerve cells. These connections are the ultimate result of coordinated developmental processes, involving direct interactions between cells for correct positioning and organization of cell layers in the brain, guidance of outgrowing axons, and the formation and shaping of synaptic contacts between them. Central to these processes are cell adhesion molecules which serve the communication and interaction between cells and thus have a key role in creating and tuning precisely-wired neural circuits.

During evolution, cell adhesion molecules have been instrumental in organizing multicellularity, thereby undergoing extreme diversifications (Abedin and King, 2008). These diversifications have been established through extensive variation of a limited number of structural amino acid motifs and protein domains. Based on structural characteristics, cell adhesion molecules have been accordingly classified in vast superfamilies such as cadherins and Ig domain cell adhesion molecules (IgCAMs). Besides these, families with fewer members and atypical adhesion domains have also been recognized, often serving more refined functions in specifying precise connections between nerve cells in specialized circuits, latrophilins (LPHNs) being one of them.

In cell adhesion, specificity is based on the nature of at least two partnering cell adhesion molecules. There is an extensive repertoire of interactions between cell adhesion molecules that forms the basis of interaction networks. Cell adhesion molecules can reside on contacting cells and interact in *trans*, or form a complex in *cis* on a single cell before partnering in *trans*. Furthermore, cell adhesion molecules can act in combination with an identical partner (homophilic complex), a different partner (heterophilic complex) or with multiple partners (multiprotein complex). While some cell adhesion molecules display strict specificity toward partners, others are more promiscuous. These properties together with the extensive diversity of cell adhesion molecules provide a shear endless combinatorial potential. It has been postulated by Thomas Südhof that the diverse and multifold protein-protein interactions constitute molecular codes that drive formation, stability and dynamics of synaptic contacts which are required for the precision of neural circuitry formation (Sudhof, 2017, 2018).

In this context, we will explore principles of neuronal cell adhesion by focusing on the family of latrophilins that display multimodal interactions. The family of latrophilins itself has already been reviewed extensively (Meza-Aguilar and Boucard, 2014). Instead, we zoom in on well-established partners of the latrophilins, particularly teneurins, fibronectin leucine-rich repeat transmembrane proteins (FLRTs) and contactins. We synopsize temporal and spatial expression profiles in combination with structural characteristics that together allow these interactions, and we discuss their functional consequences.

## Introducing Latrophilins

Latrophilin has initially been discovered as the Ca^2++^-independent receptor for alpha-latrotoxin, which is one of the toxic substances in the widow spiders’ venom (Krasnoperov et al., 1997; Lelianova et al., 1997; Sugita et al., 1998). Fast-forwarding to two decades later, the latrophilin family is now known to contain three family members (LPHN1-3), of which all three are classified as adhesion G-protein coupled receptors (GPCRs) and linked to neuronal and non-neuronal disorders including ADHD and cancer (reviewed in Meza-Aguilar and Boucard, 2014). Furthermore, it has been shown that *LPHN2* and *LPHN3* are highly expressed in specific brain areas, whereas *LPHN1* is detected at lower levels but more ubiquitously distributed throughout the brain (Sugita et al., 1998; Kreienkamp et al., 2000). Interestingly, in rodent brain *LPHN1* levels are low during early postnatal development and increase with age, whereas *LPHN2* shows the opposite pattern (Kreienkamp et al., 2000; Boucard et al., 2014). Recent data for LPHN3 show that protein expression peaks at approximately P12, when synaptogenesis is taking place (Sando et al., 2019). In contrast, peaks in *LPHN3* mRNA levels were seen very early during rat postnatal development (Kreienkamp et al., 2000) as well as at later stages in the developing mouse brain (Boucard et al., 2014). Finally, the repertoire of endogenous ligands/interacting partners of latrophilins has been expanded to four different families, namely neurexins, teneurins, FLRTs and contactins (see Figure 2A). In this review we will focus on well-described interactions with teneurins and FLRTs, and its most recently discovered interacting partner Contactin6 (CNTN6). Neurexins are not considered here, since their interaction with latrophilins has been questioned and downplayed (O’Sullivan et al., 2014; Sudhof, 2018).

## Interaction With Teneurins

In the search for ligands of latrophilins, members of the type II transmembrane teneurin family of cell adhesion molecules were the first latrophilin-interacting proteins to be identified (Silva et al., 2011). Teneurins are non-classical cell adhesion molecules that may well have functions beyond simple cell adhesion.

### Expression

**FIGURE 1:**
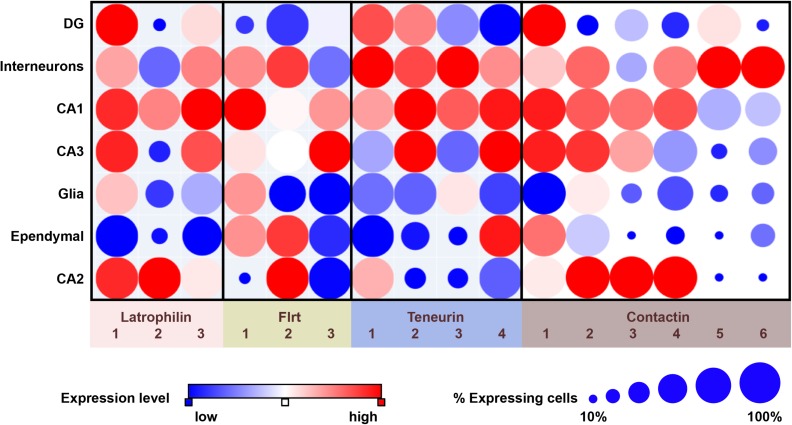
Overview of expression of latrophilins and cell-adhesion proteins of the FLRT, teneurin and contactin families in the adult mouse hippocampus. Data are from single-cell RNA sequencing and presented by dot plots using the Single Cell Portal (https://portals.broadinstitute.org/single_cell) (Habib et al., 2016). Expression levels are color-coded according to a red-blue scale (red: highest; blue: lowest). The size of dots indicates the proportion of cells that express the indicated transcript. It should be noted that single-cell RNA sequencing studies as summarized here are an excellent source for generating hypotheses, but that follow-up studies, involving for instance directed qPCR, are required when pursuing such hypotheses.

The teneurin transmembrane proteins (TENM) family members display specific developmental and topographical expression patterns in the mammalian brain. During embryonic development of the mouse central nervous system (CNS), TENM3 and TENM4 are expressed as early as E7.5, followed by expression of TENM2 around E10.5. TENM1 expression starts later, at E15.5 (Zhou et al., 2003). At that embryonic timepoint, all four teneurins are expressed in the telencephalon and diencephalon with partial overlapping expression (Bibollet-Bahena et al., 2017). Later during embryonic development, TENM2 is additionally expressed in the midbrain, as well as in the nasal cavity and TENM3 shows prominent expression in the developing whisker pad (Zhou et al., 2003; Young et al., 2013). In the adult mouse brain, this diverging – but partially overlapping – expression pattern is maintained. For instance, all four teneurins are highly expressed in the CA1 region of the hippocampus, but the CA2 region expresses TENM2, TENM3, and TENM4 at very low levels, while CA3 expresses only TENM2 and TENM4 at appreciable levels, and the dentate gyrus (DG) expresses TENM1 and TENM2, as based on single-cell transcriptomics (see Figure 1; Habib et al., 2016). Earlier papers have reported variations on this pattern (Ben-Zur et al., 2000; Zhou et al., 2003; Berns et al., 2018).

### Function

Early observations in fly already demonstrated that teneurins play a functional role in circuitry formation, specifically between olfactory receptors neurons and projection neurons in the olfactory circuitry, and also in formation of the neuromuscular junction (Hong et al., 2012; Mosca et al., 2012). Intriguingly, a mutation in TENM1 has now indeed been associated with the neurological disorder congenital general anosmia, characterized by the loss of olfaction (Alkelai et al., 2016). A more detailed understanding of teneurin functions in the developing and adult CNS is steadily emerging with the overarching concept that teneurins are required for specific targeted projections in multiple brain circuits. Currently, no functional studies have been published on the role of TENM1 in the CNS, and only one group has reported functional experiments on TENM4 (Suzuki et al., 2012). This study demonstrates that oligodendrocyte differentiation is stalled in the absence of TENM4, which results in a tremor-like phenotype in *TENM4*-null mice. Notably, Hor et al. identified three missense mutations in the human *TENM4* gene (also called *ODZ4*) that are associated with patient families displaying Essential Tremor movement disorder (see Table 1; Hor et al., 2015).

**TABLE 1:** Genetic mutations impinging upon the structure of latrophilins, teneurins, FLRTs and CNTN6 associated with human disorders.

**Gene**	**Protein**	**Mutation**	**Domain**	**Disorder**	**References**
*LPHN2*	LPHN2	L1262H	ICD	Microcephaly	Vezain et al., 2018
*LPHN3*	LPHN3	A247S	OLF	ADHD	Arcos-Burgos et al., 2010
*LPHN3*	LPHN3	R465S	HRM	ADHD	Arcos-Burgos et al., 2010; Domene et al., 2011
*LPHN3*	LPHN3	D615N	GAIN	ADHD	Arcos-Burgos et al., 2010
*LPHN3*	LPHN3	T783M	GAIN	ADHD	Arcos-Burgos et al., 2010
*LPHN3*	LPHN3	L928V	TM	ADHD	Arcos-Burgos et al., 2010; Domene et al., 2011
*TENM1*	TENM1	W1882X	YD-shell	ASD	Yuen et al., 2015
*TENM1*	TENM1	P1610L	YD shell	Anosmia	Alkelai et al., 2016
*TENM1*	TENM1	G2533S	ABD	Cerebral Palsy	McMichael et al., 2015
*TENM3*	TENM3	T695Nfs^*^5	EGF-repeat	Microphtalmia	Aldahmesh et al., 2012
*TENM3*	TENM3	V990Cfs^*^13	FN-plug	Microphtalmia	Chassaing et al., 2013
*TENM3*	TENM3	A1349G + R2563W	NHL, ABD	Microphtalmia and Intellectual Disability	Singh et al., 2019
*TENM4*	TENM4	V1138M	FN-plug	Essential Tremor	Hor et al., 2015
*TENM4*	TENM4	T1367N	NHL	Essential Tremor	Hor et al., 2015
*TENM4*	TENM4	A1442T	NHL	Essential Tremor	Hor et al., 2015; Chao et al., 2016
*FLRT3*	FLRT3	Q69K	LRR	Kallman Syndrome	Miraoui et al., 2013
*FLRT3*	FLRT3	E97G + S441I	LRR	Kallman Syndrome	Miraoui et al., 2013
*FLRT3*	FLRT3	K339R	LRR	Kallman Syndrome	Miraoui et al., 2013
*CNTN6*	CNTN6	E954V	FN4	Amyotropic Lateral Sclerosis	Daoud et al., 2011
*CNTN6*	CNTN6	G310S	Ig3-Ig4	ASD	Murdoch et al., 2015; Mercati et al., 2017
*CNTN6*	CNTN6	I529L	Ig6	ASD	Murdoch et al., 2015; Mercati et al., 2017
*CNTN6*	CNTN6	P770L	FN3	ASD, Hyperacusis	Murdoch et al., 2015; Mercati et al., 2017
*CNTN6*	CNTN6	W923X	FN4	ASD, Hyperacusis	Murdoch et al., 2015; Mercati et al., 2017

**Only those mutations with structural consequences (stop, missense, nonsense) are listed, excluding copy number variations and polymorphisms. In case of CNTN6 and ASD, only mutations that are identified in more than 1 patient are listed. X, nonsense mutation; +, compound heterozygous patients; ICD, intracellular domain; TM, transmembrane domain; ADHD, attention deficit hyperactivity disorder; ASD, autism spectrum disorder. References in round brackets demonstrate lack of association between gene and disorder.**

Considerably more functional work has been published on the role of TENM2 and TENM3 in the striatum, the visual cortex, and the hippocampus. *TENM3*-null mice have been reported to show defects in the thalamostriatal pathway, the retinal ganglial cell (RGC) to superior colliculus (SC) connections and retina to dorsal lateral geniculate nucleus (dLGN) connections (Leamey et al., 2007; Dharmaratne et al., 2012; Tran et al., 2015). Similar abnormalities were noted in the *TENM2*-null animals, where a reduced number of RGCs project to the SC and dLGN (Young et al., 2013). Antinucci et al. have demonstrated that TENM3 is also essential for connections between RGCs and the optic tectum (homologous to the mammalian SC) in fish (Antinucci et al., 2013). In fact, in absence of TENM3, the animals were less able to detect shapes or position stimuli, known as orientation-selectivity. A function for TENM3 in wiring the visual system is substantiated by human genetics research in microphthalmia disease. Patients with microphthalmia have abnormally small eyes that are functionally impaired. Thus far, two patients have been identified with homozygous mutations in the *TENM3* gene. These mutations result in a premature stop codon such that TENM3 is only partially translated (T695Nfs^*^5 and V990Cfs^*^13, see Table 1; Aldahmesh et al., 2012; Chassaing et al., 2013; Singh et al., 2019).

Most recently, an important role for TENM3 in the hippocampus has been reported (Berns et al., 2018). Berns and coworkers showed that TENM3 expression in the CA1 region and in the distal subiculum is required for connectivity between these two hippocampal regions. Using advanced mouse genetics they showed that axonal as well as dendritic teneurin is required in the connecting synapse to establish correctly-wired hippocampal circuitry. It should be noted that the much broader expression of teneurin proteins in the embryonic and adult CNS, and association with a variety of disorders (see Table 1), warrants additional functionality in other brain areas yet to be discovered.

### Structure

**FIGURE 2:**
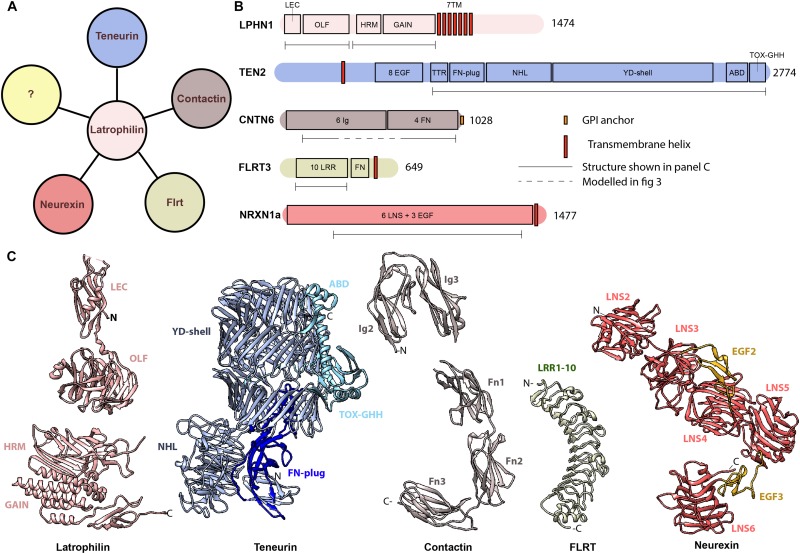
Latrophilin and its interacting protein partners **(A)** Latrophilin potentially interacts with proteins of the teneurin family, FLRTs, contactins and neurexins. The question mark (yellow) represent yet unknown interacting proteins. **(B)** Schematics of domain architecture of human latrophilin and its protein partners. **(C)** Known structures of latrophilin and interacting partners. PDB codes: 5AFB (LPHN3), 4DLQ (LPHN1), 6FB3 (TENM2), 5E55 (CNTN6), 5E5U (CNTN6), 5CMN (FLRT3), 3QCW (NRXN1). LEC, lectin domain; OLF, olfactomedin domain; HRM, hormone receptor motif; GAIN, GPCR autoproteolysis inducing; 7TM, 7 transmembrane domain; EGF, epidermal growth factor-like; FN, fibronectin; NHL, NCL-1, HT2A, and Lin-41 repeat; YD, tyrosine and aspartate-rich repeat; ABD, Antibiotic-binding domain; Ig, Immunoglobulin; LRR, leucine-rich repeat; LNS, laminin, neurexin, sex-hormone binding globulin domain.

The amino acid sequence of the extracellular region of teneurins is 59–71% identical between teneurins, and also the predicted domain organization is highly comparable. Structures of human and chick TENM2 and mouse TENM3 show that the extracellular region is folded into a large barrel-shaped structure, termed YD-shell, adorned with a beta-propeller perpendicular to the YD-shell (see Figure 2B; Jackson et al., 2018; Li et al., 2018). The barrel is sealed by a so-called fibronectin plug domain and capped by its own inward spiraling C-terminal. This C-terminal end aligns with the barrel wall and threads out through a gap in the barrel to form two additional domains, the ABD and Tox-GHH domains. So far, only the beta-propeller and the C-terminal domains have been implicated in protein-protein interactions. The barrel itself shows striking similarities with the bacterial toxin system TcB, TcC of *Y. enteromophaga* and *P. luminscencens*, and teneurin-like protein-coding genes have been identified in several other bacteria as well (Tucker et al., 2012; Jackson et al., 2018). In these bacterial systems, the barrel-containing protein is part of a much larger protein complex important for toxin injection. Although the similarity to bacterial toxin systems might lead to tempting speculations, the practical implications of the structurally similar YD-shell in mammalian teneurins remain unknown. A notable difference between the bacterial and mammalian teneurins is that in the case of mammalian teneurins, covalent dimerization is induced by a non-traditional EGF-repeat domain, that has not been observed in bacterial teneurins.

### Molecular Mechanisms: Teneurin – Latrophilin Interactions

Latrophilins are adhesion GPCRs and consist of a small intracellular domain, seven-pass transmembrane helices and a larger extracellular domain (ECD) with multiple protein motifs. The extracellular domain can be cleaved by autoproteolysis, possibly resulting in a conformational change (Hamann et al., 2015; Arac et al., 2016). The extracellular region contains the proteolytic GAIN domain and a hormone-binding (HRM) domain, followed by a glycosylated linker region and the olfactomedin-like domain as well as a rhamnose-binding lectin domain (see Figures 2B,C; Vakonakis et al., 2008; Arac et al., 2012; O’Sullivan et al., 2014). The lectin domain specifically interacts with the extracellular domain of teneurin. Although this domain is sufficient and necessary for binding, the full length ECD of latrophilin has a higher binding affinity for teneurin than lectin alone (Silva et al., 2011; Boucard et al., 2012).

Which domain on teneurin is required for the formation of this complex? Silva et al. demonstrated that the C-terminal fragment of teneurin containing only the ABD and Tox-GHH domains was able to bind full-length latrophilin. Furthermore, a deletion construct of TENM2 that is missing the ABD and Tox-GHH domains (referred to as Tox-like domain in Li et al., 2018) abrogated its capability to interact with latrophilin. Thus, the latrophilin *–* teneurin interaction might be mediated by the lectin and ABD with Tox-GHH domains, respectively.

Latrophilin is somewhat promiscuous in its teneurin partner choice. Whereas LPHN1 binds TENM2 as its highest affinity ligand (Silva et al., 2011; Boucard et al., 2012; Vysokov et al., 2016; Li et al., 2018), and vice versa (Silva et al., 2011), cellular binding assays reveal additional interactions between LPHN1 and TENM4 (Boucard). Furthermore, LPHN2 interacts with TENM2 and TENM4 (Boucard et al., 2014; Jackson et al., 2018), and LPHN3 can interact with all members of the TENM family (O’Sullivan et al., 2012; Boucard et al., 2014; Berns et al., 2018; Li et al., 2018; Sando et al., 2019). Notably, a splice insert in all three LPHNs (for mouse LPHN1, KVEQK – following Y131) as well as two splice inserts in TENM2 and TENM3 (for mouse TENM3, AHYLDKIVK following I,740 and RNKDFRH, following L1218) might both decrease binding affinity of one for another (Boucard et al., 2014; Berns et al., 2018; Li et al., 2018). The latrophilin splice insert does not affect binding to FLRT3 (this interaction is discussed in more detail below) (Boucard et al., 2014). Detailed structural information derived from X-ray crystallography and cryo-electron microscopy (cryo-EM) has provided some insight into the structural consequences of these splicing events. The splice insert in latrophilin is situated between the lectin and olfactmedin domain (Boucard et al., 2014; Jackson et al., 2015). In teneurins, the second splice insert is located in between the first and the second blade of the NHL domain, a 6 bladed beta-propeller. This insert promotes homophilic TENM2 and TENM3 interactions, with the splice insert itself forming a potential dimerization interface (Jackson et al., 2018; Li et al., 2018). Conversely, the absence of this splice insert may increase the affinity of teneurin for latrophilin.

**FIGURE 3:**
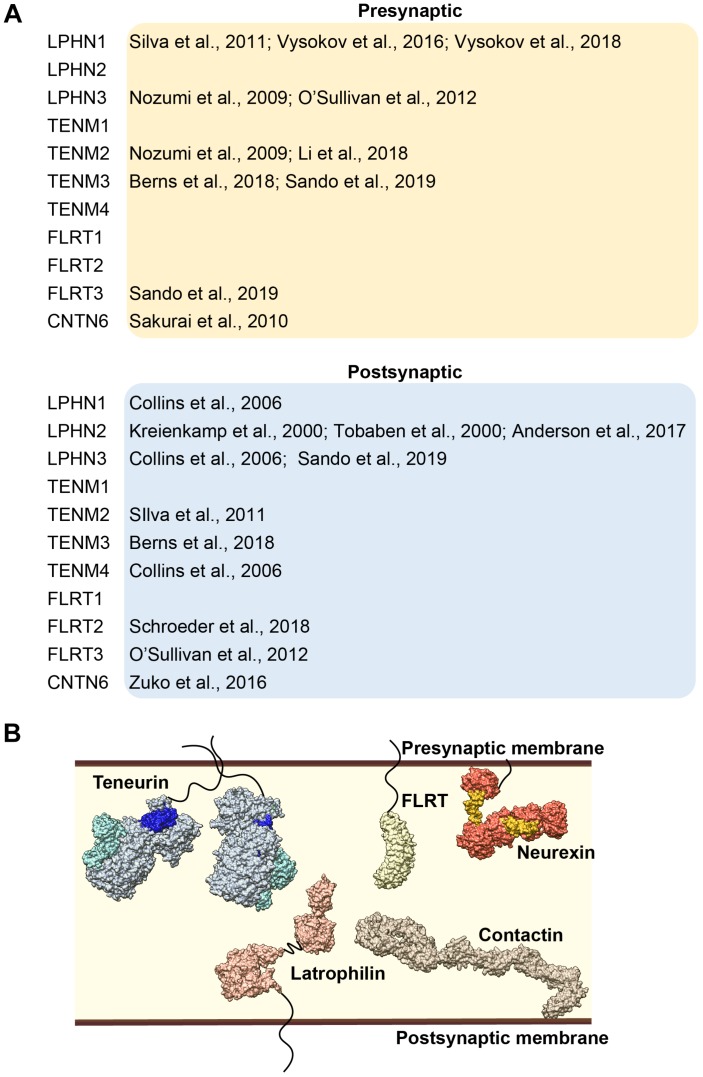
**(A)** Overview of the literature of synaptic localization of latrophilin and its interacting partners. **(B)** Model of latrophilin and its interacting partners in the synapse, not constrained by temporal or spatial expression patterns.

What about the functional consequences of latrophilin *–* teneurin complex formation? As noted previously, both latrophilin and teneurin contain transmembrane segments and complex formation has indeed been shown to occur on the membrane in NB2A cells and in HEK293T cells (Silva et al., 2011; Beckmann et al., 2013; Vysokov et al., 2016; Li et al., 2018). Furthermore, when the full-length proteins are expressed in two different cell populations, mixing and aggregation of these populations is induced, indicating that this interaction occurs in *trans* (Silva et al., 2011; Boucard et al., 2014; Berns et al., 2018; Li et al., 2018). In neurons, teneurin and latrophilin family members are both localized to the synaptic compartments. Their pre- and/or postsynaptic localization remains a topic of discussion (see Figure 3A). For instance, LPHN1 is mostly documented as pre-synaptic (Silva et al., 2011; Vysokov et al., 2016, 2018), however it is also part of the postsynaptic proteome of murine CNS (Collins et al., 2006). In addition, LPHN2 has been identified as a postsynaptic protein (Kreienkamp et al., 2000; Tobaben et al., 2000; Anderson et al., 2017), whereas LPHN3 has been observed in both compartments (Collins et al., 2006; Nozumi et al., 2009; O’Sullivan et al., 2012; Sando et al., 2019). Likewise, TENM2 and TENM3 have both been identified in presynaptic terminals (Nozumi et al., 2009; Berns et al., 2018; Li et al., 2018) as well as postsynapticcaly (Silva et al., 2011; Berns et al., 2018). Trans-synaptic interactions have already been demonstrated for the high-affinity pair Latrophilin1 and Teneurin2 (Silva et al., 2011), as well as for homophilic Teneurin3 interactions (with the splice inserts) (Berns et al., 2018). Such interactions might be instrumental for neuronal outgrowth and synapse formation, as well as synapse maintenance.

Beside the well-documented intercellular effects on cell adhesion, complex formation has also been suggested to induce intracellular signaling events. Cells that express latrophilin respond to its natural ligand alpha-latrotoxin with an increase in intracellular Ca^2+^ levels. Silva et al. have demonstrated that this response is enhanced when latrophilin-expressing cells are incubated with the ABD and Tox-GHH region of TENM2, using neuroblastoma cells (Silva et al., 2011). Also, in primary hippocampal neurons this C-terminal fragment of TENM2 induced calcium signaling (Silva et al., 2011). The relative contribution of the latrophilin *–* teneurin complex compared to TENM2 by itself remains to be tested. Furthermore, Li et al. (2018) show that the co-expression of LPHN1 and TENM2 tempers the levels of another second messenger, namely cAMP, both in experiments that mimic a *cis*-interaction as well as in a *trans* configuration set-up, using HEK293 cells. In these experiments, latrophilin1 by itself reduced cAMP levels slightly, whereas teneurin2 alone did not affect cAMP levels at all.

Most recently, *in vivo* evidence for the functional relevance of the LPHN3-TENM2 interaction has been provided by Sando et al. (2019). These authors demonstrate that a LPHN3-mutant that cannot bind TENM2, is unable to rescue a reduction in Schaffer collateral synaptic strength induced by LPHN3-deficient CA1 neurons in the hippocampus (Sando et al., 2019). Notably, a FLRT3-binding mutant of LPHN3 is also unable to rescue this phenotype (see also section Molecular Mechanisms: FLRT – Latrophilin Interactions and Discussion), indicating that TENM2 and FLRT3 binding are together required for LPHN3 function (Sando et al., 2019).

## Latrophilin – Flrt Interaction

Members of the fibronectin leucine-rich transmembrane (FLRT) family of cell adhesion proteins are a second class of binding partners of latrophilins. This subfamily consists of three members, sharing the fibronectin III domain, and are part of a much larger group of proteins that all have the leucine-rich repeat, including AMIGOs, LINGOs, LLRTMs, NLLRs, and others (Chen et al., 2006).

### Neurobiological and Developmental Functions

The original identification of the FLRTs stems from a screen for extracellular matrix proteins expressed in muscle cells (Lacy et al., 1999). Early expression and essential defects in mouse embryos indicated the important role of these proteins (Maretto et al., 2008). A defined neural role was readily assigned to FLRT2 and FLRT3. These proteins were found to be shed and to act in soluble form as repulsive cues for Unc5D-positive neurons in the mouse cortex (Yamagishi et al., 2011). Specifically, FLRT2-Unc5D interactions were further shown to direct radial migration of cortical neurons, whereas the homophilic FLRT3-FLRT3 interaction controlled tangential migration of these neurons (Seiradake et al., 2014). This dual role in corticogenesis may be a key mechanism of FLRTs in cortical folding. This is supported by the findings that accessory sulci are formed in the cortex of *FLRT1/3*-null mice, where also cortical migration defects were observed (Del Toro et al., 2017). Interestingly, species with low cortical expression of FLRT2 and FLRT3, such as man and ferret, develop folded cortices, further supporting this hypothesis. The finding that FLRTs are ligands of latrophilin and that they together instruct the development of excitatory synapses added a new dimension to the insight in the functions of FLRTs (O’Sullivan et al., 2012).

### Characteristics and Expression Profiles

Examining FLRT expression profiles in the mouse hippocampus through single-cell transcriptomics and anatomical localization databases shows that all FLRTs are expressed in the hippocampus, but with different characteristics (see Figure 1; Habib et al., 2016). FLRT1 is expressed highest in the CA1 region, less in the CA3 region and not in the CA2 and DG, while FLRT2 is expressed in the CA1 and CA2 region as well as in GABAergic interneurons (see Figure 1; Schroeder et al., 2018). FLRT3 has the most restricted hippocampal expression as it is predominantly expressed by DG and CA3 neurons (O’Sullivan et al., 2012). These patterns show that all neurons in the hippocampus express at least one type of FLRT protein. Moreover, examination of single-cell transcriptomics based on data from Habib et al. shows that co-expression of different combinations of FLRTs occurs in specific neuronal cell types (Habib et al., 2016). In the mouse cerebral cortex, the expression of FLRTs is low compared to the hippocampus. In fact, single-cell RNA seq studies indicate that FLRT1 and FLRT2 mRNAs are virtually absent in the cortex, while expression of FLRT3 is particularly expressed in a subset of GABAergic interneurons (Tasic et al., 2016).

### Structure

The amino acid sequences of all three FLRTs is quite similar, with FLRT1 and FLRT3 being the most divergent with 59% overall identity. All three FLRTs are type I single pass transmembrane proteins with a ∼100 amino acid long intracellular region and an extracellular region that compasses 10 leucine rich repeats (LRR) followed by a single fibronectin (FN) type III domain (see Figures 2B,C). The LRR domain is folded into an elongated, incurvated structure with inward facing beta strands and outwardly-extending loops (Seiradake et al., 2014). Links between FLRTs and diseases are virtually lacking as yet; there is one genome-wide association study for Kallman Syndrom (with anosmia as its most distinguishing feature) identifying three patients with mutations in FLRT3, all located in the LRR domain (see Table 1).

### Molecular Mechanisms: FLRT – Latrophilin Interactions

FLRT3 was first identified as the postsynaptic interaction partners of presynaptic LPHN3 using affinity chromatography followed by mass spectrometry (O’Sullivan et al., 2012). However, in light of the debated latrophilin localization (see section Molecular Mechanisms: Teneurin – Latrophilin Interactions), there is also some uncertainty about the *trans*-orientation of this protein interaction pair (Lu et al., 2015; see Figure 3A). On a structural level, a number of crystal structures of the FLRT – latrophilin complex reveal how the β-propeller-shaped olfactomedin domain of latrophilin is tightly bound to the incurvated surface of the LRR domain of FLRT (Lu et al., 2015; Ranaivoson et al., 2015; Jackson et al., 2016). Interestingly, in the tertiary FLRT2 – LPHN3 – Unc5D complex, a direct interaction is observed between the lectin domain of LPHN2 and Unc5D, mediated by a salt bridge between residue E105 in LPHN2 and R156 in Unc5D (Jackson et al., 2016). FLRTs interact with LPHNs with some specificity for certain family members: FLRT1 and FLRT3 interact with all three LPHNs, while FLRT2 interacts with LPHN3 (O’Sullivan et al., 2012; Jackson et al., 2015, 2016).

Insight into the neurobiological significance of the LPHN-FLRT interaction has been limited so far to one particular high-affinity partnership, namely FLRT3-LPHN3 (O’Sullivan et al., 2012). In neuronal cultures, reduction in FLRT or LPHN3 expression, or interference with the FLRT-LPHN interactions resulted in a decrease of the density of glutamatergic synapses (O’Sullivan et al., 2012). In a similar fashion, reduction of FLRT expression *in vivo* reduced the number of perforant-path synapses and the strength of glutamatergic transmission (O’Sullivan et al., 2012). Moreover, Sando and coworkers have demonstrated that a reduced number of Schaffer collateral synapses in LPHN3 transgenic mice is not rescued by FLRT3 or TENM2 binding-mutants of LPHN (Sando et al., 2019). Instead, they postulate that LPHN3 requires simultaneous FLRT3 and TENM2 interactions for its synaptogenic functions (Sando et al., 2019). Together, these findings may be prototypical for the neurobiological potential of FLRT interactions, however, more extended studies on these functions have not been published yet. An argument to suspect that FLRT interactions play a more generic role in shaping morphology and function of brain circuits comes from the work of de Wit group, showing that an array of cell adhesion proteins of the Leucine-rich repeat (LRR) family, including FLRT2, regulates synapse structure and function of CA1 pyramidal neurons (Schroeder et al., 2018).

## Latrophilin – Contactin6 Interaction

The most recent protein found to interact with LPHNs is Contactin6 (CNTN6). CNTN6 is classified as an IgCAM and belongs together with its five paralogs to the mammalian contactin IgCAM subfamily. In CNTN6 pull-down experiments, latrophilin-family member LPHN1 appeared as one of the most prominent proteins bound to CNTN6 (Zuko, 2015, 2016a). This interaction was demonstrated to occur in *cis*, using cell-binding and cell-aggregation assays. In view of the identified CNTN6 *–* LPHN1 interaction, we here focus on CNTN6 and only discuss other CNTN members in that context.

### Expression

CNTN6 expression is strongly regulated during mouse development with peak expression in early postnatal stages, as revealed by expression of a *LacZ* gene inserted in the mouse *CNTN6* locus (Takeda et al., 2003). Brain regions with strong X-Gal staining were the accessory olfactory bulb, the anterodorsal thalamus, layer V of the cerebral cortex, inferior colliculus and the cerebellum. Further analyses based on *in situ* hybridization of CNTN6 mRNA confirmed CNTN6 expression in these areas, but also indicated a wider expression involving other areas, in particular the hippocampus and multiple cortical layers. Regarding the hippocampal area, CNTN6 expression is found in the CA1 and the hilus of the dentate gyrus (Zuko et al., 2016b). Sakurai et al. also observed CNTN6-immunoreactivity in the subiculum, the stratum lacunosum–moleculare of the CA1 region and confirmed the expression of CNTN6 in the hilus of the dentate gyrus (Sakurai et al., 2010). Examining data from single-cell RNA sequencing partly confirms these observations and indicates that the CNTN6-positive cells in the hilus may be interneurons (see Figure 1; Habib et al., 2016). Analyses of the mouse cortex showed presence of CNTN6 mRNA and protein in layers II/III and confirmed expression in layer V (Zuko et al., 2016b), with single-cell RNA sequencing revealing CNTN6 expression in multiple cell types (Tasic et al., 2016). Specifically, highest levels of CNTN6 transcripts were found in layer V pyramidal neurons, and vasointestinal peptide (VIP)- and somatostatin (Sst)-expressing interneurons in the motor cortex (Tasic et al., 2016).

CNTN6 is highly expressed in the cerebellum and displays differential expression over lobules in the adult brain. For instance, CNTN6 is highly expressed in subpopulations of granule cells and in the molecular layer of lobule 1 to the rostral half of lobule 9, but expression in the distal region of lobules 9 and 10 is weak (Takeda et al., 2003). During the development of the cerebellum, the *CNTN6* gene is first expressed in the Purkinje cells of lobules 9 and 10 and is followed by expression in the internal granule cells of all lobules. At P5 and thereafter, CNTN6 immunoreactivity was observed in the developing molecular layer and granule cell layer but not in Purkinje cells in lobule 23.

### Function

CNTN1 and CNTN2 are the prototypical members of the CNTN family. For over 20 years these proteins are known as important components in neuron-glia interactions and formation of the nodes of Ranvier (Peles and Salzer, 2000; Ascano et al., 2012). CNTN1 and CNTN2 have been demonstrated to regulate neuronal migration, axon guidance and the organization of specific subdomains in the nodes of Ranvier through *cis*- and *trans*-interactions with distinct cell adhesion molecules (Mohebiany et al., 2014). CNTN1 and CNTN2 have been taken as examples of the principal functions and mechanisms of action for the other members of this family. However, the other members lack the essential functions in neuron-glia interactions in myelination. Although much less well characterized, these CNTN family members have prominently come forward in genetic studies on neuropsychiatric developmental disorders (see Table 1; Guo et al., 2012; Nava et al., 2014; Huang et al., 2017; Oguro-Ando et al., 2017). Further, phenotypes in null mutant mice have indicated functions of these CNTNs in the developing and mature brain (Shimoda and Watanabe, 2009).

Specifically CNTN6 has been recognized as a potential player in a number of neurobiological processes, mostly through human genetics, loss-of-function studies in mice and gain-of-function studies *in vitro*. Human genetics has shown the association of CNTN6 variants, mostly copy number variations, with neuropsychiatric conditions including autism spectrum disorder, hyperacusis, anorexia nervosa and Tourette syndrome (Huang et al., 2017; Oguro-Ando et al., 2017). In animal models, there is only a single study available at the behavioral level, reporting mild phenotypes in *CNTN6*-null mice (Takeda et al., 2003). These mice exhibited impaired motor coordination indicating cerebellar deficits. This finding may well relate to the neuroanatomical observations of developmental cerebellar expression of CNTN6 (see section Expression).

Several studies have reported neuroanatomical phenotypes of *CNTN6*-null mice. Sakurai and coworkers showed that in the hippocampus of *CNTN6*-null mice CNTN6 appears to affect glutamatergic but not GABAergic synapses based on reduced expression of VGLUT1 and VGLUT2 and unaltered expression of VGAT (Sakurai et al., 2010). Similarly, CNTN6 is involved in the development of glutamatergic neurons in the cerebellum. In particular, CNTN6 was shown to colocalize with presynaptic marker VGLUT1 in parallel fibers that synapse on Purkinje cells (Sakurai et al., 2009). In the cortex of the same mouse strain, a modest shift in the numbers of subtype-specific projection neurons and interneurons in the visual cortex was observed (Zuko et al., 2016b). Furthermore, Ye et al. noted misorientation of the apical dendrite of pyramidal neurons in the visual cortex, particularly in layer V, in *CNTN6*-null mice (Ye et al., 2008). Combined loss-of-function of CNTN6 and one of its interaction partners CHL1, a neural IgCAM, dramatically aggrevated this dendritic phenotype. Interestingly, it was demonstrated that both CNTN6 and CHL1 interacted with protein tyrosine phosphatase α (PTPα, PTPRA), which is highly abundant in the brain (Kaplan et al., 1990; Ye et al., 2008). The authors proposed a signaling complex in which PTPα is downstream of CHL1 and CNTN6 and which regulates apical dendrite projections in the developing cortex (Ye et al., 2008).

Additional data have supported a role of CNTN6 in neuronal outgrowth and survival. For instance, the formation and terminal branching of the corticospinal tract is delayed in *CNTN6*-null mice, and neurite growth and neuronal survival is impaired in *CNTN6*-null mice with cerebral ischemia, aggrevating ischemic damage (Huang et al., 2011, 2012). Furthermore, a neuronal outgrowth role was also revealed in another condition of neurotrauma, namely spinal cord injury. In these mice, the regrowth of corticospinal axons was stimulated in the absence of CNTN6 protein or when CNTN6 was downregulated by shRNA (Huang et al., 2016).

From a molecular perspective, CNTN6 protein has also been characterized as a ligand for the receptor protein NOTCH. CNTN6 binds to NOTCH1, induces the cleavage and nuclear translocation of the NOTCH intracellular domain and subsequently, drives the expression of NOTCH1 target genes such as *HES1* (Cui et al., 2004). CNTN6-mediated Notch activation was proposed to serve the differentiation of oligodendrocytes (Cui et al., 2004).

### Structure

All CNTNs are composed of six Ig domains followed by four fibronectin-III (FnIII) domains, anchored to the membrane via a GPI-linker. Several of these domains have now been solved structurally using X-ray crystallography, but no full-length structure of the extracellular segment is yet available. The structure of the first four Ig domains has been determined for chicken CNTN2, human CNTN2 and mouse CNTN4. In all three cases, the Ig domains fold into a typical horsehoe-like configuration, in which the first Ig domain contacts the fourth Ig domain, and the second Ig domain interacts with the third Ig domain (see Figures 2B,C; Freigang et al., 2000; Mortl et al., 2007; Bouyain and Watkins, 2010). More recently, a structure of CNTN3 spanning the fifth Ig domain until the second FnIII domain, as well as the structure of the first three FnIII domains of all six contactins, was determined (Nikolaienko et al., 2016). Together, these structures reveal how a sharp bend between the second and third FnIII domain might induce a parallel orientation of the extended Contactin structure toward the cell surface. Mutations in the third and fourth FNIII domains as well as in the Ig3-Ig4 and Ig6 domains have been found in patients with ASD and hyperacusis, supporting the notion that all these structural domains contribute essentially to the functional properties of CNTN6 protein (see Table 1; Mercati et al., 2017). While CNTN6 has no transmembrane or intracellular regions, CNTN6 can still participate in synaptic signaling through multiple protein interactions. From *in vivo* and *in vitro* studies, a model for *cis* interactions of CNTN6 proteins has been put forward (Ye et al., 2008; Ye et al., 2011). In this model, CNTN6 is part of an axonal complex with CHL1 and PTPσ (PTPRS), a protein related to PTPα. The latter interaction is supported by crystallographic studies providing the structural basis of the interaction of CNTN6 with PTPγ (PTPRG), another member of the PTP family (Bouyain and Watkins, 2010; Nikolaienko et al., 2016). In addition, CNTNs might also form homodimers that could result in interactions *in trans* (Huang et al., 2016). Thus, involvement of CNTN6 in neurobiological processes might require multimodal *cis* and *trans* interactions, that we are now only starting to unravel.

### Molecular Mechanisms: Contactin – Latrophilin Interactions

How can CNTN6 complex with latrophilins? Using cellular aggregation assays, a *cis*-LPHN1-CNTN6 complex is more strongly supported than a complex in *trans* (Zuko et al., 2016a). Thus far, no experiments have been performed to map the interacting domains or residues. As such, it is difficult to predict the architecture of the complex. For a more distant family member of the contactins, namely Neurofascin, it has been show that its FN domain interacts with gliomedin, containing multiple olfactomedin domains (Labasque et al., 2011). Thus, by comparison we could speculate that the FN domains of CNTNs are likely to interact with the olfactomedin domain of LPHN1. On the other hand, FN domains can also interact with lectins, which suggests the possibility of an interaction between the FN domains of CNTNs with the lectin domain of LPHN1 (Praetorius et al., 2001). In addition, the FN domains of CNTN5 have been assigned as interaction sites with amyloid precursor-like protein-1 (APLP1) (Shimoda et al., 2012). Clearly, additional data are needed to understand the structural basis of this complex.

Functionally, it has been shown that CNTN6 and LPHN1 indeed modulate each other’s activity. In neuronal cultures, LPHN1 overexpression resulted in an increase of apoptosis, which was blocked by co-expression of CNTN6 (Zuko et al., 2016a). Notably, overexpression of CNTN6 by itself had no effect on neuronal morphology or survival. In contrast, in cultured neurons, as well as in cortical tissue derived from *CNTN6*-null mice, enhanced apoptosis was observed (Zuko et al., 2016a). This was counteracted by shRNA-mediated LPHN1 knockdown. These results indicate a context-dependent functional interaction between CNTN6 and LPHN1. Future work is needed to resolve in greater detail how this interactions controls apoptosis in neuronal cultures, as well as in the *in vivo* setting.

Future research might reveal functional interactions in additional brain areas, since directed *in situ* hybridization experiments with LPHN1 and CNTN6 revealed co-expression in the thalamic nuclei, cortical layer V, hippocampal area CA1, and in the granular cell axons of the molecular layer in the cerebellum, pointing to the possibility that in these regions functional interactions can occur (Malgaroli et al., 1989; Zuko et al., 2016a).

## Discussion

*Trans*-synaptic interactions between cell adhesion molecules have been identified as essential elements for synapse formation and plasticity. These processes are ruled by combinatorial codes of cell adhesion molecules, which is illustrated by the complexity and multifold interactions of proteins encoded by the neurexin genes (Sudhof, 2017). The mechanisms uncovered for neurexins are pivotal when considering functions of multimodal interactions of latrophilin as reviewed here.

The neurexin family of cell-adhesion proteins consists of thousands of isoforms of transmembrane proteins encoded by three separate genes. Neurexins are expressed by neurons all over the nervous system and their expression is already initiated during brain development before synaptogenesis occurs. Neurexins have a presynaptic localization and have been extensively characterized for their central organizing roles in synapse formation, maintenance and plasticity (Sudhof, 2017, 2018).

We postulate here an analogous organizing role for latrophilins, although the extent of this role is more limited than that of neurexins in view of the more restricted expression of *LPHN2* and *LPHN3*. Furthermore, co-expression of latrophilins with established partners suggests that specific combinations exist in small subsets of neurons only, rather than in global neuronal populations as is the case for neurexins. Some of these combinations may be more widely occurring, like interactions with teneurins, than with others. This argues against a general role of latrophilin interaction networks, but rather points toward a role in refining synaptic properties of specific subtypes of neurons, requiring specific combinations of proteins. The current data start to reveal what this refinement may imply and what neuronal subtypes employ latrophilin interaction networks.

Here, we have reviewed temporal and spatial expression patterns of its protein partners teneurins, FLRTs and CNTN6 (see Figure 3B). Discrete expression in time together with cell-type specificity determines which interacting partners are available for complex formation. For instance, during late embryonic brain development, interactions with LPHN2 are less likely to play an important role due to very low to absent protein expression. Beyond temporal and spatial availability, specificity of interactions is also generated by splicing events, exemplified by the interaction between latrophilin and teneurin. Furthermore, although no data are available yet on post-translational modifications (PTMs) in latrophilins or its partners, PTMs in general are well-known to determine binding specificity and affinity of interactions. A type of PTM that is especially of importance for extracellular interactions is modification by glycans, also known as glycosylation. In fact, N-linked glycans are now known to be highly abundant and particularly variable in synaptic proteins (Trinidad et al., 2013). For instance, latrophilins are predicted to be decorated with as many as 7 N-linked glycans and harbor an O-linked sugar-rich region in between the HRM and OLF domains (O’Sullivan et al., 2014). Future studies are needed to test their importance in protein-protein interactions. The exact location and identity of these glycans can be mapped using a combination of mass spectrometry and structural biology techniques. An elegant example of how N-glycans impact cell adhesion complexes is the presence of three glycosylated residues in SynCAM that regulate adhesion (Fogel et al., 2010).

What about the functional consequences of latrophilin interactions? Evidence for a functional role of latrophilin-centered protein networks comes from an *in vivo* transgenic study, as well as cellular assays on the LPHN3 – FLRT3 – TENM2 network (Sando et al., 2019). Neither of the latrophilin mutants that are incapable of FLRT3 or TENM2 binding could rescue the latrophilin-induced decrease in Schaffer collateral synaptic strength *in vivo* (Sando et al., 2019). The requirement of both binding sites for latrophilin function indicates that multimodality might be essential in latrophilin-instructed synaptogenesis. In addition, the finding that LPHN3 mutations associate with ADHD indicates an important functional role in humans (see Table 1). Also for teneurins, FLRTs and CNTN6 human genetic data indicate specific defects to be associated with these interactors. It will be essential to determine which of these relate to partnering to latrophilins, and what other, still unknown partners are involved in these phenotypes.

## Outlook

For integration of spatial and temporal expression patterns, splicing events and PTMs of latrophilin and its protein partners, high-resolution imaging while maintaining temporal and spatial information is desired. Whereas previous insights mostly involved freeze substitution electron microscopic tomography, techniques such as cryo-electron microscopy and cryo-electron tomography are now expected to produce high resolution structures in cellular contexts (Lucic et al., 2005; Zuber et al., 2005; High et al., 2015; Perez de Arce et al., 2015). The follow-up, understanding of the precise function of such protein networks will still be an enormous endeavor, but we may expect that on the way we will be able to recognize novel neurobiological mechanisms that are inherent to the latrophilins.

## Author Contributions

Both authors listed have made a substantial, direct and intellectual contribution to the work, and approved it for publication.

## Conflict of Interest Statement

The authors declare that the research was conducted in the absence of any commercial or financial relationships that could be construed as a potential conflict of interest.
